# Editorial: International Partnerships for Strengthening Health Care Workforce Capacity: Models of Collaborative Education

**DOI:** 10.3389/fpubh.2018.00002

**Published:** 2018-02-07

**Authors:** Jeanne Mahoney Leffers, Jennifer G. Audette

**Affiliations:** ^1^Community Nursing, University of Massachusetts Dartmouth, North Dartmouth, MA, United States; ^2^Physical Therapy, University of Rhode Island, Kingston, RI, United States

**Keywords:** partnerships, collaboration, education, sustainability, health workforce capacity, global health

**Editorial on the Research Topic**

**International Partnerships for Strengthening Health Care Workforce Capacity: Models of Collaborative Education**

According to the World Health Organization, by 2035 the availability of health care workers will fall short of meeting the need by a figure of 12.9 million (1). This shortage disproportionately impacts low resource settings where great disparities in health outcomes exist. To address this serious problem, many governmental, non-governmental, and academic organizations develop educational partnerships to strengthen capacity among health care workers where the need is the greatest. However scholars are critical of some of the approaches utilized in the growing participation in international service (2–4), since these approaches often fail to develop equitable partnerships. Reports suggest that effective partnerships should be culturally appropriate, collaborative, bidirectional, and sustainable with strong leadership from the host partners (5–7). This E-Book, *International Partnerships for Strengthening Health Care Workforce Capacity: Models of Collaborative Education*, offers significant examples of effective models of international collaborative education to build health workforce capacity to improve health care outcomes, and provide guidance for what true partnerships can and should look like.

The manuscripts include examples from a variety of professional disciplines and geographic settings. Further, the focus of the projects range from addressing the needs of pregnant women, newborns, children, and adults with acute and chronic health needs for both emergent and restorative care. Initially, more than 40 manuscripts were submitted for this specific Research Topic (RT). Of the 38 deemed appropriate to the RT, 31 have been accepted for publication. Those chosen for publication provide a fairly equal distribution of medical subspecialties, nursing and midwifery, and physical and occupational therapy. The partnerships represent a broad geographic spectrum, with papers reporting projects in Cuba, Haiti, Nicaragua, Guatemala, Ecuador, Guyana, Dominican Republic, Suriname, Nigeria, Kenya, Uganda, Sudan, Malawi, Tanzania, Ghana, Ethiopia, Rwanda, Nepal, Laos, Cambodia, Nepal, India, Turkey, Serbia, and the Solomon Islands.

From the collection of 31 published manuscripts from the RT, we selected 18 that represent perspectives, community case study, curriculum, instruction and pedagogy and evaluation types across academic, non-governmental organization and other global partnership forms. The published manuscripts highlight various elements of partnerships, with a strong focus upon collaboration, mutual planning and capacity building. Some of the examples are in the early stages of planning and partnership development, while others highlight initiatives that have been sustained over many decades. Approximately one-third of the manuscripts report academic partnerships between higher resource and low resource settings, another third are from Health Volunteers Overseas sponsored programs and the final third are from other global health organizations such as Save the Children International, the Global Health Service Partnership, and Kybele.

Every manuscript included in this Research Topic addresses partnerships, but some directly address many of the central elements of partnership formation or continuance, such as the importance of developing trust between partners, respect for local culture, mutuality for planning and outcomes, capacity building for health care workforce, bidirectional programming, and empowerment of host partners to lead and sustain programs. Pinner and Kelly provide an overview of the establishment and development of projects designed to build collaborative partnerships with academic and health institutions across the globe over the 30-year history of Health Volunteers Overseas (HVO). Other long-term partnerships discussed by Mbalinda et al.; Cech and Alvarado; and Panigua-Avila et al. offer insights about how the partnership is structured, showing the shared roles of the collaborating partners, achievements over time, and as models of how partnerships can build sustainability or broaden their collaboration.

Mbalinda et al. focus upon an HVO collaboration in nursing education that has been sustained for more than 17 years to the mutual benefit of the HVO volunteer nurses as well as the academic and clinical nurses in Kampala, Uganda. Cech and Alvarado report on a 20-year collaboration to improve health, education and advocacy for children with disabilities in Ecuador while Panigua-Avila et al. describe a 12-year partnership between three universities in Guatemala and the University of Pennsylvania in the US, whose aim is to develop mutually beneficial exchanges to build clinical, educational and leadership skills to improve health for underserved communities in both locations. O’Sullivan et al., discuss key elements of partnership formation through their case study of an Ireland–Uganda initiative designed to explore joint educational opportunities for students in both settings to strengthen educational and research capacity for global health, disability and rehabilitation.

Other manuscripts by Yu et al.; Buser; Leader et al.; and Koster et al. focus upon priority health needs in countries with scarce resources and limited health care workforce. Specifically, Leader et al.; Koster et al.; and Baysinger et al. address the high rates of infant and child mortality in resource scarce settings that are a serious problem in many locations globally. Eckerle et al. highlight educational innovations to address this health issue.

A common critique of global aid programs (4, 7, 8) is the number of programs based in high resource settings, such as the US, that send health professionals to low resource settings without acknowledging or realizing that services and programs already exist. Efforts are then duplicated without collaboration. In fact, these situations may undermine or interfere with local efforts already underway. With this in mind, Eckerle et al. offer an example of a partnership between Kamazu Central Hospital in Malawi and three US-based institutions that strengthen health systems through a coordinated approach. The program forges stronger expertise among all partners and avoids the risk of duplication that is so common in global health programs. Additionally, Koster et al. report a partnership between three US institutions and the Hopital Ste. Damien-Nos Petits Freres and Soeurs in Haiti that reduces redundancy and builds collaboration by limiting the visiting partners from the US to a smaller group of clinicians.

International collaborations are also used to develop, redesign, and upgrade training curricula. This often serves two purposes: it advances the status of the profession and often has the effect of increasing the number of participants and results in more graduates. This is particularly important in low resource settings where dire shortages of providers persist. Such a partnership is described in the paper by Audette et at. The manuscripts by Footer et al. and Pascal et al. describe the importance of empowering and developing leadership skills in young professionals, many of whom are among the first of their discipline to practice in their country.

Education is a vital key to strengthening capacity of health care workers, and many of the manuscripts offer educational strategies, describing a variety of pedagogical approaches. Although mentorship is not a new pedagogy, Catton reports how Save the Children International has had success in an educational partnership in Laos to increase the skills and capacity of the midwifery workforce using a mentorship strategy. Pediatric specialists McConnell et al. offer a case example of a Telehealth program that uses technology to sustain a partnership for ongoing education in Nicaragua for pediatric nurses. To build capacity for health, Leader et al. used two well-developed and standardized educational programs: *Helping Babies Breathe* and *Essential Care for Every Baby*. The programs employ a train-the-trainer model, in a partnership between a broad range of entities: the Ministry of Health, the Dominican Pediatric Society, the Pan American Health Organization, UNICEF, multiple international and Dominican NGOs, and individuals from three different US medical institutions. The project partners were able to educate 17 trainers who then reached nearly 350 providers.

Henker et al. highlight a simulation course developed at the request of the Angkor Hospital for Children in Siem Reap, Cambodia. While the partnership has continued for many years, this innovative educational training highlights the successes of technical training in settings where advanced technology for health education and clinical care is not available. A number of educational initiatives include program evaluation to support the educational innovation as reported by Buser, Yu et al., Leader et al., McConnell et al., and Henker et al.

Paramount to any successful collaboration is sustainability. Partnerships where a focus on sustainability has been a key element from the start are included in several of the manuscripts. Issues such as maintaining strong partnerships and keeping a program flexible and innovative over the course of time are elements of the manuscripts by Leader et al., Cech and Alvarado, and Mbalina et al. The nature and complexity of international partnerships, paired with the difficulties inherent in measuring programmatic and learner outcomes result in challenges in evaluating long-term outcomes. Many of the articles report attempts to meet that challenge by creating programs where student assessment leads to outcome measurement. Stuart Shor et al. provide evaluation data for the first 3 years of the Global Health Service Partnership (GHSP), a unique collaboration between the Peace Corps, SEED and PEPFAR in Malawi, Uganda, and Tanzania. Almost 100 nurse and physician educators provided training to more than 8,000 partner trainees, practicing health professionals and faculty over the 3-year period. Significant findings emphasize the importance of culturally appropriate and locally tailored educational strategies. Others such as Pinner and Kelly have built systematic program assessment processes into their models.

We are pleased to be able to share so many creative, interesting, and diverse models of global health initiatives. It is exciting to reflect on how this work has evolved over the past decades and to think about all of the potential there is for even more innovative work in the future. In closing, it seems appropriate to include the words of Paul Farmer: “with rare exceptions, all of your most important achievements on this planet will come from working with others—or, in a word, partnership” (9).

## Author Contributions

JL was lead author for the editorial submitted for this RT. JA contributed to and helped to edit the editorial submission.

## Conflict of Interest Statement

The authors declare that the research was conducted in the absence of any commercial or financial relationships that could be construed as a potential conflict of interest.
